# Benzo[*a*]fluoren-11-one

**DOI:** 10.1107/S1600536808020989

**Published:** 2008-07-12

**Authors:** Di Sun, Geng-Geng Luo, Su-Yuan Xie, Rong-Bin Huang, Lan-Sun Zheng

**Affiliations:** a Department of Chemistry, Xiamen University, Xiamen 361005, People’s Republic of China; b State Key Laboratory for Physical Chemistry of Solid Surfaces, Xiamen University, Xiamen 361005, People’s Republic of China

## Abstract

The mol­ecule of the title compound, C_17_H_10_O, is nearly planar, the largest deviation from the mean plane being 0.06 Å. The crystal structure is governed by π–π inter­actions, with centroid–centroid distances ranging from .559 to 3.730 Å.

## Related literature

For related literature, see: Banik *et al.* (2006 ▶); Huang *et al.* (1997 ▶); Peng *et al.* (2001 ▶); Streitweiser & Brown (1988 ▶); Xie *et al.* (2001 ▶).
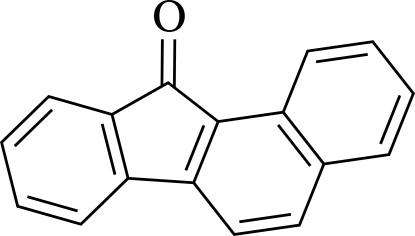


## Experimental

### 

#### Crystal data

C_17_H_10_O
*M* *_r_* = 230.25
Monoclinic, 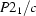

*a* = 9.3852 (4) Å
*b* = 7.1165 (3) Å
*c* = 16.8809 (7) Å
β = 99.278 (5)°
*V* = 1112.72 (8) Å^3^
*Z* = 4
Mo *K*α radiation
μ = 0.08 mm^−1^
*T* = 173 (2) K
0.55 × 0.20 × 0.20 mm

#### Data collection

Oxford Gemini S Ultra diffractometer
Absorption correction: multi-scan (*CrysAlis RED*; Oxford Diffraction, 2007 ▶) *T*_min_ = 0.955, *T*_max_ = 0.983
4736 measured reflections
2134 independent reflections
1433 reflections with *I* > 2σ(*I*)
*R*_int_ = 0.034

#### Refinement

*R*[*F* ^2^ > 2σ(*F* ^2^)] = 0.047
*wR*(*F* ^2^) = 0.094
*S* = 1.01
2134 reflections
163 parameters
H-atom parameters constrained
Δρ_max_ = 0.15 e Å^−3^
Δρ_min_ = −0.17 e Å^−3^

### 

Data collection: *CrysAlis CCD* (Oxford Diffraction, 2007 ▶); cell refinement: *CrysAlis RED* (Oxford Diffraction, 2007 ▶); data reduction: *CrysAlis RED*; program(s) used to solve structure: *SHELXS97* (Sheldrick, 2008 ▶); program(s) used to refine structure: *SHELXL97* (Sheldrick, 2008 ▶); molecular graphics: *ORTEP-3* (Farrugia, 1997 ▶); software used to prepare material for publication: *SHELXL97*.

## Supplementary Material

Crystal structure: contains datablocks I, global. DOI: 10.1107/S1600536808020989/dn2363sup1.cif

Structure factors: contains datablocks I. DOI: 10.1107/S1600536808020989/dn2363Isup2.hkl

Additional supplementary materials:  crystallographic information; 3D view; checkCIF report

## Figures and Tables

**Table 1:** π–π Interactions (Å)

	Centroid–centroid	Interplanar distance	Slippage
*Cg*1⋯*Cg*1^i^	3.683	3.46	1.26
*Cg*1⋯*Cg*2^i^	3.627	3.48	0.98
*Cg*1⋯*Cg*4^ii^	3.559	3.38	1.06
*Cg*2⋯*Cg*3^i^	3.730	3.49	1.23
*Cg*3⋯*Cg*4^ii^	3.667	3.38	1.31

Symmetry codes: (i) 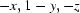; (ii) 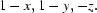

**Fig. 1.**
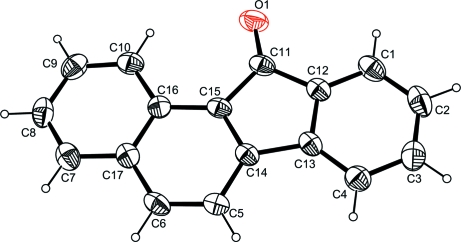
ORTEP Molecular view of compound I. Thermal ellipsoids are drawn at the 50% probability level. H atoms are represented as small spheres of arbitrary radii.

## supplementary crystallographic information

### Comment

Benzo[*a*]fluoren-11-one, C_17_H_10_O, (Scheme), can be readily synthesed by oxidation of the corresponding hydrocarbon with different oxidants, such as NaBiO_3 _(Banik *et al.*, 2006), or by Friedel-Crafts ring closure reaction(Streitwieser *et al.*, 1988). But its crystal structure determination has not been carried out yet. During the past decade, our group has used various non-organic methods, such as high-voltage electric discharge in liquid (Huang *et al.*, 1997), vaporized (Xie *et al.*, 2001) chloroform and CCl_4_ and solvothermal reaction (Peng *et al.*, 2001) to generate and trap a family of perchlorinated fullerene fragments. Recently in our low pressure premixed benzene-oxygen combustion system, we generated the compound, C_17_H_10_O, and isolated it. We report here the synthesis and crystal structure of the compound.

The title compound, C_17_H_10_O, is built up from four fused rings. The whole molecule is nearly planar with the largest deviations from the mean plane being 0.06Å (Fig. 1). The crystal packing is governed by π-π interactions (Table 1).

### Experimental

The compound was prepared in low pressure pre-mixed benzene-oxygen flames. The premixed flames conditions for the soot production as the following range: atom C/O ratio:1–2; combustion chamber pressure: 350torr. The soot collected from the water-cooled coping was extracted with toluene using an ultrasonic bath under room temperature, the resulting dark-brown solution was separated and purified by multi-stage high-preformance liquid chromatography(HPLC), finally we obtained one of fractions contained pure C_17_H_10_O. The red single crystals suitable for X-ray diffraction crystallized from toluene at room temperature. The product was analyzed by Atmospheric-Pressure Chemical Ionization(APCI) mass spectrometry(negative mode). The molecular peak appeared at a mass/charge ratio of 230.

### Refinement

All H atoms were placed geometrically and treated as riding with C—H distances of 0.95 Å and *U*_iso_= 1.2U_eq_(C).

### Crystal data

**Table:**

C_17_H_10_O	*F*_000_ = 480
*M**_r_* = 230.25	*D*_x_ = 1.374 Mg m^−^^3^
Monoclinic, *P*2_1_/*c*	Mo *K*α radiation λ = 0.71073 Å
Hall symbol: -P 2ybc	Cell parameters from 1792 reflections
*a* = 9.3852 (4) Å	θ = 2.7–32.6º
*b* = 7.1165 (3) Å	µ = 0.08 mm^−^^1^
*c* = 16.8809 (7) Å	*T* = 173 (2) K
β = 99.278 (5)º	Prism, red
*V* = 1112.72 (8) Å^3^	0.55 × 0.20 × 0.20 mm
*Z* = 4	

### Data collection

**Table:**

Oxford Gemini S Ultra diffractometer	2134 independent reflections
Radiation source: fine-focus sealed tube	1433 reflections with > 2σ
Monochromator: graphite	*R*_int_ = 0.034
*T* = 173(2) K	θ_max_ = 26.0º
ω scans	θ_min_ = 3.6º
Absorption correction: empirical (using intensity measurements)(CrysAlis RED; Oxford Diffraction, 2007)	*h* = −11→10
*T*_min_ = 0.955, *T*_max_ = 0.983	*k* = −8→8
4736 measured reflections	*l* = −20→20

### Refinement

**Table:**

Refinement on *F*^2^	Secondary atom site location: difference Fourier map
Least-squares matrix: full	Hydrogen site location: inferred from neighbouring sites
*R*[*F*^2^ > 2σ(*F*^2^)] = 0.047	H-atom parameters constrained
*wR*(*F*^2^) = 0.094	*w* = 1/[σ^2^(*F*_o_^2^) + (0.0409*P*)^2^] where *P* = (*F*_o_^2^ + 2*F*_c_^2^)/3
*S* = 1.01	(Δ/σ)_max_ < 0.001
2134 reflections	Δρ_max_ = 0.15 e Å^−^^3^
163 parameters	Δρ_min_ = −0.17 e Å^−^^3^
Primary atom site location: structure-invariant direct methods	Extinction correction: none

### Special details

**Table:**

Experimental. Empirical absorption correction using spherical harmonics, implemented in SCALE3 ABSPACK scaling algorithm. CrysAlis RED (Oxford Diffraction, 2007)
Geometry. All e.s.d.'s (except the e.s.d. in the dihedral angle between two l.s. planes) are estimated using the full covariance matrix. The cell e.s.d.'s are taken into account individually in the estimation of e.s.d.'s in distances, angles and torsion angles; correlations between e.s.d.'s in cell parameters are only used when they are defined by crystal symmetry. An approximate (isotropic) treatment of cell e.s.d.'s is used for estimating e.s.d.'s involving l.s. planes.
Refinement. Refinement of *F*^2^ against ALL reflections. The weighted *R*-factor *wR* and goodness of fit *S* are based on *F*^2^, conventional *R*-factors *R* are based on *F*, with *F* set to zero for negative *F*^2^. The threshold expression of *F*^2^ > σ(*F*^2^) is used only for calculating *R*-factors(gt) *etc*. and is not relevant to the choice of reflections for refinement. *R*-factors based on *F*^2^ are statistically about twice as large as those based on *F*, and *R*- factors based on ALL data will be even larger.

### Fractional atomic coordinates and isotropic or equivalent isotropic displacement parameters (Å^2^)

**Table:**

	*x*	*y*	*z*	*U*_iso_*/*U*_eq_	
O1	0.20866 (13)	0.18498 (17)	−0.07292 (8)	0.0441 (4)	
C1	−0.02937 (18)	0.4405 (3)	−0.16653 (10)	0.0378 (5)	
H1A	−0.0396	0.3189	−0.1900	0.045*	
C2	−0.11989 (19)	0.5866 (3)	−0.19725 (11)	0.0438 (5)	
H2A	−0.1930	0.5651	−0.2422	0.053*	
C3	−0.10410 (18)	0.7630 (3)	−0.16279 (11)	0.0411 (5)	
H3A	−0.1668	0.8614	−0.1846	0.049*	
C4	0.00180 (17)	0.7996 (3)	−0.09676 (11)	0.0356 (4)	
H4A	0.0125	0.9214	−0.0735	0.043*	
C5	0.26877 (16)	0.7875 (2)	0.05511 (10)	0.0302 (4)	
H5A	0.2280	0.9100	0.0512	0.036*	
C6	0.38358 (17)	0.7465 (2)	0.11267 (10)	0.0333 (4)	
H6A	0.4220	0.8422	0.1492	0.040*	
C7	0.56835 (17)	0.5255 (3)	0.17902 (10)	0.0368 (5)	
H7A	0.6054	0.6201	0.2164	0.044*	
C8	0.63221 (18)	0.3530 (3)	0.18308 (11)	0.0395 (5)	
H8A	0.7138	0.3288	0.2229	0.047*	
C9	0.57847 (17)	0.2119 (3)	0.12911 (11)	0.0397 (5)	
H9A	0.6246	0.0927	0.1321	0.048*	
C10	0.46019 (17)	0.2432 (3)	0.07188 (11)	0.0346 (4)	
H10A	0.4238	0.1450	0.0361	0.041*	
C11	0.18804 (17)	0.3497 (2)	−0.05773 (10)	0.0313 (4)	
C12	0.07505 (15)	0.4757 (2)	−0.10158 (9)	0.0291 (4)	
C13	0.09063 (15)	0.6536 (2)	−0.06621 (10)	0.0282 (4)	
C14	0.21217 (16)	0.6458 (2)	0.00190 (9)	0.0272 (4)	
C15	0.27059 (16)	0.4674 (2)	0.00659 (9)	0.0275 (4)	
C16	0.39179 (16)	0.4204 (2)	0.06560 (9)	0.0278 (4)	
C17	0.44778 (16)	0.5651 (3)	0.11987 (10)	0.0304 (4)	

### Atomic displacement parameters (Å^2^)

**Table:**

	*U*^11^	*U*^22^	*U*^33^	*U*^12^	*U*^13^	*U*^23^
O1	0.0524 (8)	0.0301 (8)	0.0491 (8)	−0.0072 (6)	0.0059 (6)	−0.0097 (7)
C1	0.0367 (9)	0.0464 (12)	0.0317 (9)	−0.0128 (9)	0.0093 (8)	−0.0059 (10)
C2	0.0361 (10)	0.0628 (15)	0.0302 (9)	−0.0109 (10)	−0.0011 (8)	0.0009 (11)
C3	0.0332 (9)	0.0504 (13)	0.0389 (10)	0.0002 (9)	0.0032 (9)	0.0091 (11)
C4	0.0329 (9)	0.0376 (10)	0.0369 (10)	−0.0036 (8)	0.0076 (8)	0.0004 (9)
C5	0.0322 (8)	0.0281 (9)	0.0315 (9)	−0.0046 (8)	0.0090 (8)	−0.0040 (9)
C6	0.0380 (9)	0.0355 (10)	0.0271 (9)	−0.0106 (8)	0.0072 (8)	−0.0086 (9)
C7	0.0354 (9)	0.0485 (12)	0.0271 (9)	−0.0076 (9)	0.0070 (8)	−0.0004 (10)
C8	0.0317 (9)	0.0562 (14)	0.0297 (10)	−0.0007 (9)	0.0021 (8)	0.0106 (10)
C9	0.0378 (10)	0.0390 (11)	0.0445 (11)	0.0035 (9)	0.0130 (9)	0.0117 (10)
C10	0.0354 (9)	0.0340 (11)	0.0366 (10)	−0.0079 (8)	0.0128 (8)	0.0000 (9)
C11	0.0331 (9)	0.0311 (10)	0.0319 (9)	−0.0098 (8)	0.0113 (8)	−0.0029 (9)
C12	0.0288 (8)	0.0343 (10)	0.0257 (9)	−0.0069 (8)	0.0092 (8)	−0.0011 (9)
C13	0.0244 (8)	0.0370 (11)	0.0245 (8)	−0.0073 (8)	0.0078 (7)	−0.0012 (9)
C14	0.0256 (8)	0.0316 (10)	0.0260 (9)	−0.0074 (7)	0.0087 (7)	−0.0007 (9)
C15	0.0294 (8)	0.0276 (9)	0.0277 (9)	−0.0080 (8)	0.0111 (7)	−0.0011 (9)
C16	0.0261 (8)	0.0320 (10)	0.0272 (9)	−0.0047 (7)	0.0095 (7)	0.0041 (9)
C17	0.0282 (9)	0.0385 (11)	0.0258 (9)	−0.0061 (8)	0.0083 (8)	0.0015 (9)

### Geometric parameters (Å, °)

**Table:**

O1—C11	1.222 (2)	C7—C17	1.412 (2)
C1—C12	1.371 (2)	C7—H7A	0.9500
C1—C2	1.389 (3)	C8—C9	1.395 (3)
C1—H1A	0.9500	C8—H8A	0.9500
C2—C3	1.381 (3)	C9—C10	1.367 (2)
C2—H2A	0.9500	C9—H9A	0.9500
C3—C4	1.393 (2)	C10—C16	1.411 (2)
C3—H3A	0.9500	C10—H10A	0.9500
C4—C13	1.380 (2)	C11—C15	1.486 (2)
C4—H4A	0.9500	C11—C12	1.491 (2)
C5—C6	1.361 (2)	C12—C13	1.397 (2)
C5—C14	1.397 (2)	C13—C14	1.484 (2)
C5—H5A	0.9500	C14—C15	1.380 (2)
C6—C17	1.421 (2)	C15—C16	1.426 (2)
C6—H6A	0.9500	C16—C17	1.422 (2)
C7—C8	1.362 (3)		
			
C12—C1—C2	118.49 (18)	C8—C9—H9A	119.6
C12—C1—H1A	120.8	C9—C10—C16	120.40 (17)
C2—C1—H1A	120.8	C9—C10—H10A	119.8
C3—C2—C1	120.36 (17)	C16—C10—H10A	119.8
C3—C2—H2A	119.8	O1—C11—C15	127.75 (16)
C1—C2—H2A	119.8	O1—C11—C12	126.62 (16)
C2—C3—C4	121.42 (18)	C15—C11—C12	105.62 (14)
C2—C3—H3A	119.3	C1—C12—C13	121.30 (16)
C4—C3—H3A	119.3	C1—C12—C11	130.30 (16)
C13—C4—C3	117.91 (17)	C13—C12—C11	108.39 (13)
C13—C4—H4A	121.0	C4—C13—C12	120.51 (15)
C3—C4—H4A	121.0	C4—C13—C14	131.32 (16)
C6—C5—C14	118.63 (16)	C12—C13—C14	108.15 (15)
C6—C5—H5A	120.7	C15—C14—C5	121.40 (15)
C14—C5—H5A	120.7	C15—C14—C13	109.10 (15)
C5—C6—C17	122.13 (16)	C5—C14—C13	129.49 (16)
C5—C6—H6A	118.9	C14—C15—C16	121.37 (15)
C17—C6—H6A	118.9	C14—C15—C11	108.72 (14)
C8—C7—C17	120.77 (17)	C16—C15—C11	129.91 (15)
C8—C7—H7A	119.6	C10—C16—C17	118.86 (15)
C17—C7—H7A	119.6	C10—C16—C15	124.34 (15)
C7—C8—C9	120.38 (16)	C17—C16—C15	116.79 (15)
C7—C8—H8A	119.8	C7—C17—C6	121.51 (16)
C9—C8—H8A	119.8	C7—C17—C16	118.79 (16)
C10—C9—C8	120.77 (17)	C6—C17—C16	119.69 (14)
C10—C9—H9A	119.6		

### Table 1 π–π Interactions (Å)

**Table:**

	Centroid–centroid	Interplanar distance	Slippage
Cg1···Cg1^i^	3.683	3.46	1.26
Cg1···Cg2^i^	3.627	3.48	0.98
Cg1···Cg4^ii^	3.559	3.38	1.06
Cg2···Cg3^i^	3.730	3.49	1.23
Cg3···Cg4^ii^	3.667	3.38	1.31

Symmetry codes: (i)  -x, 1-y, -z; (ii) 1-x, 1-y, -z.
